# A Practical Guide to Conducting Dose-Response Meta-Analyses in Epidemiology

**DOI:** 10.5964/meth.14733

**Published:** 2025-06-30

**Authors:** Huan Jiang, Jürgen Rehm, Charlotte Probst, Alexander Tran, Shannon Lange, Laura Llamosas-Falcón

**Affiliations:** [1] Institute for Mental Health Policy Research, Centre for Addiction and Mental Health, Toronto, ON, Canada.; [2] Dalla Lana School of Public Health, University of Toronto, Toronto, ON, Canada.; [3] Campbell Family Mental Health Research Institute, Centre for Addiction and Mental Health, Toronto, ON, Canada.

**Keywords:** epidemiology, dose response, risk curves, methodology, dose-response meta-analysis

## Abstract

Dose-response relationships between continuous risk factors and disease outcomes are necessary for understanding the risks related to different levels of exposure. Dose-response risk curves can lead to more targeted public health messaging, prevention efforts, and policy implementation. Meta-analyses are often used to combine statistical results from different studies and can be used to model dose-response relationships. However, several challenges are encountered when performing dose-response meta-analysis, such as having heterogeneous reference categories, inconsistent measures of risk, and determining the most accurate shape of the curve. In this paper, we propose a three-step process for estimating dose-response relationships via meta-analysis, which involves: 1) harmonizing the measures of risk, 2) homogenizing the reference category, and 3) selecting meta-regression models. We use data obtained from a systematic review on the dose-response relationship between alcohol consumption and the risk of chronic liver disease to provide an example of the proposed process.

Dose-response relationships, often represented as dose-response curves, describe the magnitude of risk as a function of exposure—that is, in epidemiology, they represent the relationship between a continuous risk factor, such as amount of alcohol consumed, and a particular health outcome (for example, see GBD 2020 Alcohol Collaborators, 2022; Rehm, Rovira, et al., 2021). Knowing the dose-response relationship is central to determining the hazardous levels of an exposure, which allows for the creation of targeted public health messaging, prevention efforts, and policy measures (Keyes & Galea, 2016). For example, whether a risk factor has a J-shape or a monotonic relationship with an outcome, or whether the dose-response relationship is linear has important implications for selecting both individual-level (e.g., increased education and health literacy) and population-level (e.g., policy implementation) interventions.

Meta-analyses are often used to combine statistical results from different studies and using regression models to integrate dose-specific data enables modeling of dose-response relationships (Zhang et al., 2018). However, inaccurate modeling of these relationships due to violating model assumptions, or poor aggregation methods across heterogeneous studies can severely impact the accurate estimation of the level of risk related to a given level of exposure. Thus, it is crucial that relationships are correctly modeled, as the utilization of inaccurate dose-response relationships by decision- and policymakers can have significant repercussions for public health.

A significant challenge that is faced when synthesizing epidemiological studies is the heterogeneity in reference points, as they often vary across studies. For instance, in the case of substance use, the three most common reference categories are: (i) lifetime abstainers, (ii) current abstainers (including both former users and lifetime abstainers), and (iii) the lowest consumption category (for discussion, see Klatsky, 2008; Rehm et al., 2008). Moreover, some studies examine multiple risk factors, each conceptualized in a different way, further complicating the synthesis process. Additional challenges encountered when performing a dose-response meta-analysis include inconsistent measures of risk—for instance, some studies will provide relative risks (RR) while others will provide odds ratios (OR) or hazards ratios (HR)—as well as determining the most accurate shape of the curve. Here, we propose a three-step process for estimating dose-response relationships via meta-analysis, which involves: 1) harmonizing the measures of risk, 2) homogenizing the reference category, and 3) selecting the most appropriate meta-regression models. Each step is explained in detail to guide the estimation of dose-response relationships via meta-analysis.

Additionally, a practical example is provided, using data on the relationship between alcohol consumption and chronic liver disease (for an overview, see Rehm, Patra, et al., 2021) obtained from a systematic review (PROSPERO registration number CRD42022299680, for results see Llamosas-Falcón et al., 2024). This example demonstrates the generation of pooled dose-response curves for relationships between continuous behavioral risk factors and health outcomes. The demonstration concludes with a sensitivity assessment, comparing the proposed method to alternative approaches.

## Method

### Step 1: Preparation of Dose-Response Data, and Harmonizing Measures of Risk

Before a dose-response meta-analysis can be performed, the available literature must be systematically searched and data from all available studies meeting a pre-specified list of inclusion and exclusion criteria must be extracted. The Preferred Reporting Items for Systematic Reviews and Meta-Analyses (PRISMA) (Page et al., 2021) should be used as a reporting guideline. With respect to dose-response analyses specifically, selecting a standard reference point requires careful consideration to ensure comparability across studies. In the example here, studies using lifetime abstainers, current abstainers, or the lowest category of alcohol consumption as their reference category were all retained and included (for inclusion criteria, see Supplementary Material A in Jiang et al., 2025a; for research strategy, see Supplementary Material B in Jiang et al., 2025a).

One of the first challenges often encountered in a dose-response meta-analysis is the inconsistency in reporting measures of risk. Both OR and RR refer to the ratio of risk of an event occurring in an exposed group versus the risk of it occurring in a comparison group, while the OR refers to the ratio of odds (the probability of the event is divided by 1 minus the probability of the event) of an event in the exposed group versus the odds of the event in the comparison group. When the outcome is rare—i.e. less than 10%—the OR approximates the RR (Ranganathan et al., 2015). Given that an OR can be inflated when the incidence rate of the outcome is high, the RR is considered to be the more appropriate estimate under such circumstances (Zhang & Yu, 1998) and can be obtained by dividing the risk (incidence proportion, rate) in the exposed group by the risk in the comparison group. In cohort studies, where ORs are calculated using logistic regression, the OR should be recalculated as an RR, using the following formula [Formula 1]:

(Formula 1) $$\text{Relative}\;\text{risk}=\frac{\text{odds}\;\text{ratio}}{1-p_{0}+p_{0}\times \text{odds}\;\text{ratio}}$$

Where *p*_0_ is the baseline risk, which is the incidence or prevalence of the outcome of interest in the non-exposed group (Zhang & Yu, 1998).

Additionally, when the outcome is a mortality outcome, HRs can be reported. If the outcome probability is low enough by the end of follow-up, i.e. less than 10%, RR and HR are approximately equal (VanderWeele, 2020). However, the HR can diverge significantly from the RR if the outcome is common during the follow-up. In fact, the RR reduction is a decreasing function of time, and is therefore related to a particular follow-up time. These differences between HR and RR should be carefully considered when harmonizing measures of risk.

The notation and data (see Jiang et al., 2025b) required for a dose-response meta-analysis for a generic study are displayed in Table 1, using the four papers included in our practical example, each of which used a different comparison group.

### Step 2: Selection of a Standard Reference Point and the Re-Scaling of All Pairwise Comparisons

To synthesize studies using heterogeneous reference categories in their dose-response analyses, a crucial first decision is to select a standard reference point. Preference should be given to the counterfactual scenario that is most meaningful in terms of risk assessment (Murray & Lopez, 1999). For dose-response relations in alcohol use, the standard reference point is lifetime abstention (for further reasoning for alcohol, see Rehm et al., 2001). Current abstainers for most outcomes are not a suitable reference since current abstainers are composed of lifetime abstainers and former drinkers. Part of the latter group may have stopped drinking for health reasons or may have an elevated risk for the outcome studied compared to lifetime abstainers or both. Using this group as the reference group could lead to inaccurate estimates of dose-response effects if a protective effect is falsely assumed for low levels of exposure due to the increased risk in the abstainers who were previously exposed (for previous studies, see Shaper et al., 1988).

Therefore, before performing the dose-response analysis, all RRs must be compared against the same reference category, to reduce heterogeneity between effects, and to increase the comparability of the data used in the statistical analyses. For studies using current abstainers as the reference group, successive steps can be used to change the reference category: first, separate the risks of former drinkers from those of lifetime abstainers. This separation can be done based on the subset of studies that used lifetime abstainers as the reference category where both categories (lifetime abstainers and former drinkers) and their respective risks are explicitly reported (e.g., Roerecke & Rehm, 2011). This subset of studies can be used to determine: 1) the RR of former drinkers compared to lifetime abstainers, and 2) the estimated proportion of both groups in studies using current abstainers as reference. This information then can be used to estimate a risk for lifetime abstainers with current abstainers as the reference group using the following formula [Formula 2]:

(Formula 2) $$\frac{RR_{\text{former}\;\text{drinkers}}}{RR_{\text{curent}\;\text{abstainers}}}\times P_{\text{former}\;\text{drinkers}}+\frac{RR_{\text{lifetime}\;\text{abstainers}}}{RR_{\text{current}\;\text{abstainers}}}\times P_{\text{lifetime}\;\text{abstainers}}=1$$

Or equivalently [Formula 3],

(Formula 3) $$\frac{RR_{\text{former}\;\text{drinkers}}}{RR_{\text{lifetime}\;\text{abstainers}}}\times P_{\text{former}\;\text{drinkers}}+P_{\text{lifetime}\;\text{abstainers}}=\frac{RR_{\text{current}\;\text{abstainers}}}{RR_{\text{lifetime}\;\text{abstainers}}}$$

For example, when the proportions of former drinkers and lifetime abstainers are 30.82% and 69.18%, respectively, the RR of former drinkers to lifetime abstainers is 1.8076. Using Formula 2, one can calculate that the RR of lifetime abstainers to current abstainers as 0.8007(= 1/(0.3082*1.8076 + 0.6918)). As such, in the next step, one can re-calculate the RR of the interested level or dose of the risk factor compared to lifetime abstainers based on the studies that have current abstainers as reference group by multiplying the RR compared to current abstainers by 1.2489(= 1/0.8007) [Formula 4].

(Formula 4) $$\frac{RR_{\text{interested}\;\text{risk}\;\text{level}}}{RR_{\text{lifetime}\;\text{abstainers}}}=\frac{RR_{\text{interested}\;\text{risk}\;\text{level}}}{RR_{\text{current}\;\text{abstainers}}}\times \frac{RR_{\text{current}\;\text{abstainers}}}{RR_{\text{lifetime}\;\text{abstainers}}}$$

For those studies using the lowest consumption category as the reference group (no risk estimates for lifetime or current abstainers, for some examples of papers included in the analyses see Bellentani et al., 1997; Innes et al., 2022; Schwartz et al., 2013), the RR can be estimated from a meta-regression model based on the rest of the studies (i.e., those with lifetime abstainers as reference category and those with modified risk estimates originally using current abstainers as reference category). The “*metareg*” function from the R metafor package (Viechtbauer, 2010) can be used to build a linear meta-regression model using R command: mod = *metareg(x, ~dose, intercept =, method.tau =*), where the R object x can be generated from R command *metagen* that pre-calculate the desired effect size for each study so that all have the same format. From the regression model, the corresponding risk of the lowest alcohol consumption (e.g., 7), compared to lifetime abstainers, can be estimated by using R command *predict(mod, 7, transf=exp)* along with their standard errors and confidence intervals. The RR values for other levels of consumption, compared to lifetime abstainers, are calculated by adding together the RR values for the lowest consumption and those referencing it as the baseline category. Through this calculation, studies that employ diverse reference categories are consolidated and a dataset using lifetime abstainers as the reference category is compiled. Table 2 presents the four papers included in Table 1 with the RR estimated based on the methods described above. In addition, the analysis of Llamosas-Falcón and colleagues. (2021) provides an illustration of this procedure.

The techniques described above, can also be used for tobacco or drug use (i.e., adjusting analyses to be compared to lifetime non-smokers/non-users, when provided with current non-smokers/non-users serving as the reference category).

### Step 3: Performing Dose-Response Analysis

There are two sub-steps in the dose-response analysis. First, RRs or log-transformed RRs from all selected studies are pooled using a meta-analytic multivariate Restricted Maximum Likelihood (REML) random-effects model (DerSimonian & Laird, 1986), as implemented through “*rma.mv*” function from package “metafor” (Viechtbauer, 2010) of R software version 4.0.5 (R Core Team, 2022). Given the dependency introduced by extracting several effect sizes from one study, along with between-studies variability and within study variability, three-level meta-analytic models can be applied by feeding “*rma.mv*” with a “*list()”* structure (see Supplementary Material C in Jiang et al., 2025a for code examples, Assink & Wibbelink, 2016; Fernández-Castilla et al., 2020). Using this function, various meta-regression models (e.g., linear on the logarithmic scale, quadratic, restrictive cubic splines, polynomial) can be tested to identify the one that best fits the data based on model fitting statistics such as R-Squared, Akaike information criterion (AIC) and Bayesian information criterion (BIC) statistics (Harrell, 2010). This process aims to determine the optimal dose-response relationship between the risk factor (e.g., alcohol consumption, as in the example used here) and the health outcome (whether it be a mortality and/or morbidity outcome) (see Supplementary Material C in Jiang et al., 2025a for a code sample using R). The choice of model—whether linear on the logarithmic scale, quadratic, restricted cubic splines, or polynomial models—depends on several factors, including the nature of the data, the biological relationship between risk factor and health outcome, and the need for interpretability. It is suggested to begin by visualizing the data and incrementally test more complex models while checking residuals and model fitting statistics such as AIC, BIC and R-Squared at each step. Specifically, when using log-transformed RRs, a value of 1 will be assigned to the exposure variable in the reference category, since the resulting logarithm of the RR in that category equals zero (log 1). As a result, no intercept models should be used when specifying the argument “*mods*”.

Second, control variables, such as sociodemographic variables (sex, age, country, socioeconomic status), are included in the dose-response model as covariates, based on scientific rationale. For instance, in our example, covariates and their interaction terms were included into the model step-wise (Whittingham et al., 2006), and only those that were associated with effect size differences or explained heterogeneity in the data were kept in the final meta-regression model. The resulting model can then be used to estimate beta coefficients, as well as predict and plot the predictions using *predict()* and *regplot()* functions. It is worth noting that predictions should only be made for the range of exposure level covered by the studies. For instance, in the case of alcohol and most disease and mortality outcomes, the range falls below 150 grams pure alcohol per day. This is especially important for log-transformed RR, as they may reach to biologically implausible values at higher levels of alcohol consumption (for general considerations see Gmel et al., 2013). This issue is illustrated in the practical example of the dose-response meta-analysis presented below.

For a graphical summary of the three-step process, see Supplementary Material D in Jiang et al. (2025a).

### Sensitivity Analysis

To test the robustness of our proposed methodology, two sensitivity analyses were conducted for the relationship between alcohol consumption and chronic liver disease. For the first sensitivity analysis, no adjustments were done to the reference categories, allowing us to investigate the impact of our proposed adjustments. For the second sensitivity analysis, the alternative approach of a one-stage dose-response meta-analysis using a REML random-effects estimator was conducted (Crippa et al., 2019, Crippa & Orsini, 2016). The one-stage dose-response meta-analysis uses a linear mixed model to estimate dose-response relationships across multiple studies simultaneously. However, in contrast to our proposed approach, performing a one-stage dose-response meta-analysis requires more detailed data per study. This can be more challenging to implement and interpret due to its statistical complexity, and may result in fewer studies to pool, thus reducing statistical power. The package *dosresmeta* was used for the second sensitivity analysis.

## Results

### Example: The Dose-Response Relationship Between Alcohol Use and Chronic Liver Disease

We conducted a systematic review of the literature on the relationship between alcohol consumption and chronic liver disease using the databases PubMed/Medline and Embase from inception to March 08, 2023. A total of 49 studies fulfilled our inclusion criteria with 4,712,042 participants and 15,030 cases of chronic liver disease (see Supplementary Material E for the study selection flow chart and Supplementary Material F for the studies included in the meta-analysis, both in Jiang et al., 2025a). For an overview of the characteristics of a sample see Llamosas-Falcón et al. (2024). The inclusion criteria and search strategy can be found in the Supplementary Materials A and B in Jiang et al. (2025a). Given chronic liver diseases are considered as a rare disease with around 18 per 100,000 population in 2019 (Wu et al., 2024), the OR and RR were considered to be equivalent. We excluded studies that were not published as full reports and studies with not enough data to compute the risk. In our systematic review, among the 49 studies that were included, a total of eight studies had lifetime abstainers, 16 studies had current abstainers, and 25 studies had the lowest category of alcohol consumption as their reference category. A table was compiled, similar to Table 1, with all included papers (for characteristics and risk estimates of the selected papers see Supplementary Material F in Jiang et al., 2025a and Llamosas-Falcón et al., 2024).

Formula 2 was then used to estimate the risk of lifetime abstainers with current abstainers as the reference group. The RRs of each level or dose of the risk factor compared to lifetime abstainers were obtained by multiplying the inverse of the risk by the RRs using current abstainers as the reference. The RR values for other levels of alcohol consumption, compared to lifetime abstainers, were calculated by adding together the RR values for the lowest alcohol consumption, predicted by a meta-regression model, and those referencing it as the baseline category.

In the final step, different meta-regression models (e.g., linear in the logarithmic scale, quadratic, restrictive cubic splines, polynomial) were tested and we selected the quadratic model since it provided best fit the data based on AIC and BIC statistics. Control variables (sex, cause of liver cirrhosis (LC), quality score, country—U.S. vs. others, and outcomes—mortality vs. morbidity) and their effects were tested using a Wald-type test with the quadratic model. Non-significant predictors were removed to obtain a final model that included only significant co-variables. The dose-response relationship was then estimated from the model coefficients, where the covariates were assigned certain values to reflect different background, e.g., different country, cause of liver cirrhosis, and quality score. Table 3 shows the resulting quadratic functions with beta coefficients for both women and men. Given that the quadratic function peaked at certain levels, such as 82.5 grams for women and 97.5 grams for men, we assumed the risk would plateau after reaching those dose thresholds. Therefore, the dose-response relationships were formed in a piece-wise function, as illustrated in Table 3. Those functions were then used to predict the outcome with the dose ranging from 0 to 150 grams, based on the range of exposed levels as given in the original studies.

**Figure 1:**
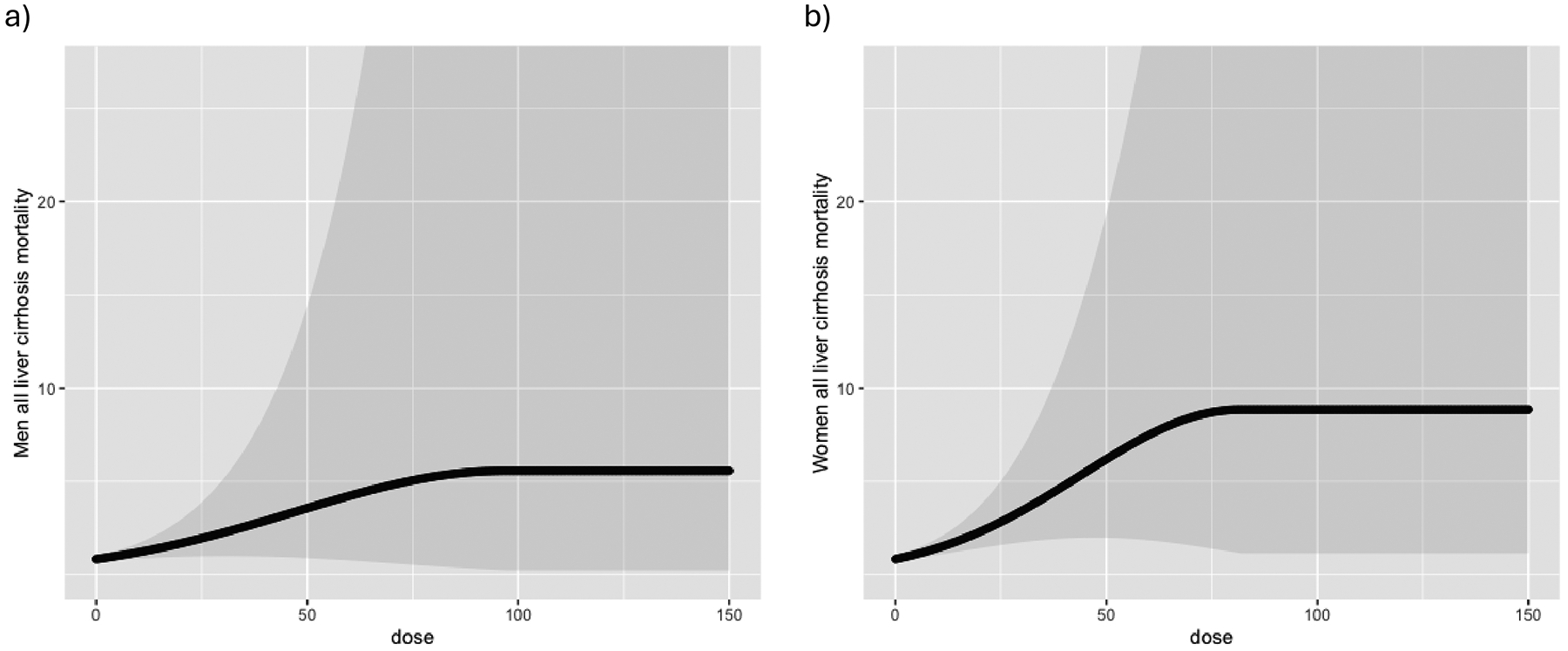
Dose-Response Relationship Between Alcohol Consumption and Chronic Liver Disease by Sex for All Causes of Chronic Liver Disease: a) Men and b) Women

Figure 1 displays the identified dose-response relationship: an asymptotic curve that reaches its maximum at 82.5 grams per day in the case of women and 97.5 grams per day in the case of men, and then stabilizes. In line with the dose-response function, it showed an elevated risk of chronic liver disease up to the point where the risk stabilizes (Kamper-Jørgensen et al., 2004; Sørensen, 1989). If we don’t assume that the risk stops increasing after reaching certain doses, the curve will continue to rise steadily, resulting in an exponential increase in the RR that is not biologically plausible.

### Sensitivity Analysis

**Figure 2:**
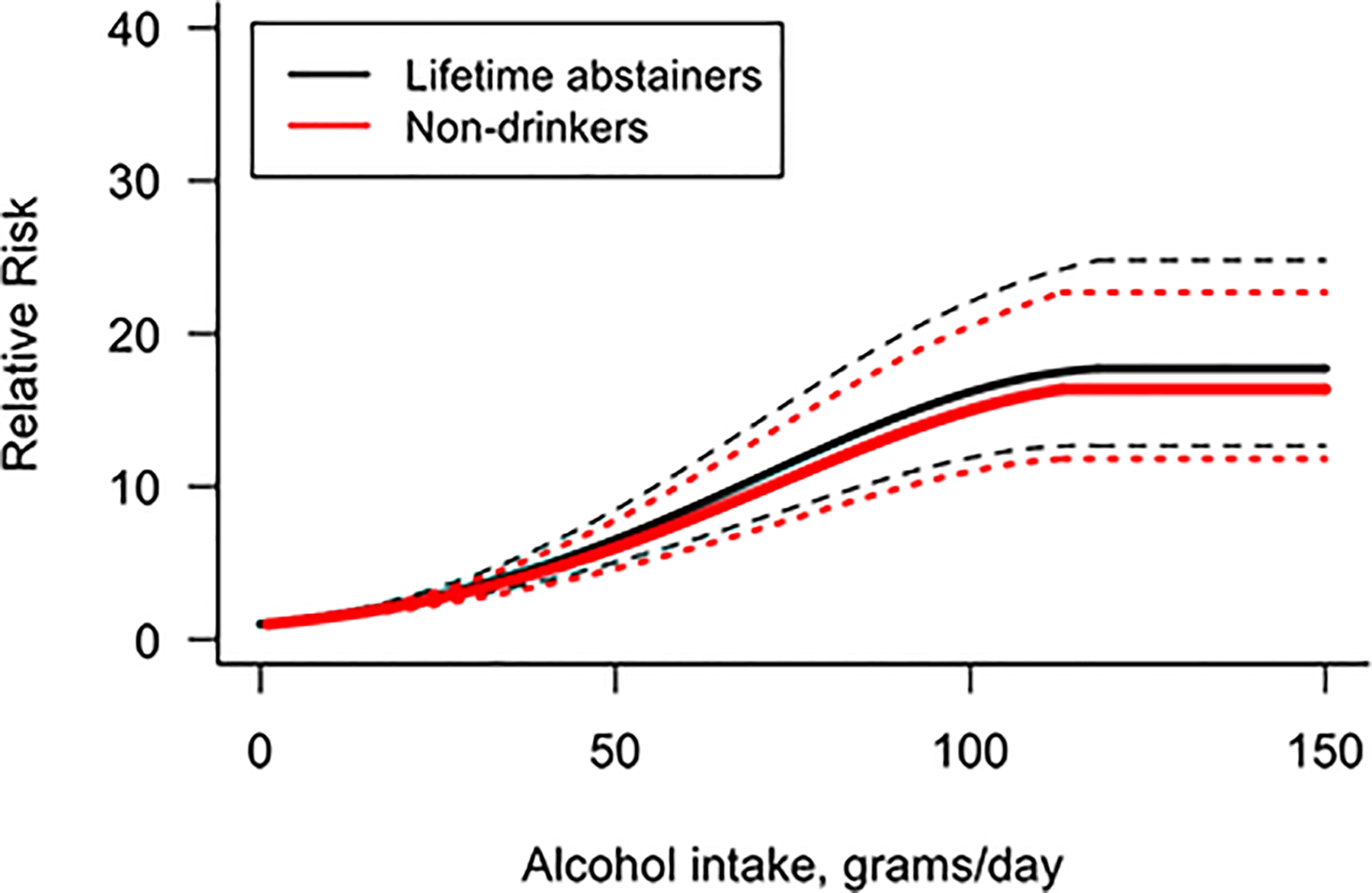
Dose-Response Relationship Between Alcohol Consumption and Al- Cause Chronic Liver Disease When Using Current Non-Drinkers as Reference Category Compared to Our Main Analysis

For each of the sensitivity analyses, we had to exclude some papers. Papers that used a reference point other than zero were excluded in the first sensitivity analysis (resulting in *n* = 21 studies being excluded), and papers that did not report the number of cases per category were excluded for the second sensitivity analysis (resulting in *n* = 26 studies being excluded). The two sensitivity analyses were conducted using data for both sexes combined, as the power was reduced for the sex-specific analysis. In the first sensitivity analysis, we used a combined reference category of lifetime abstainers and former alcohol consumers (i.e., non-drinkers). The analysis identified a quadratic dose-response relationship. Compared to the results obtained using the proposed methodology, using this combined reference category appeared to underestimate the risk relationship between average alcohol volume consumed per day and chronic liver disease (Figure 2). In addition, we obtained a slightly better model fit, as per the AIC and BIC values, when using our proposed reference category adjustments (AIC: 238.59 and BIC: 249.32), compared with the model when no adjustments were done (AIC: 238.74 and BIC: 249.46).

**Figure 3:**
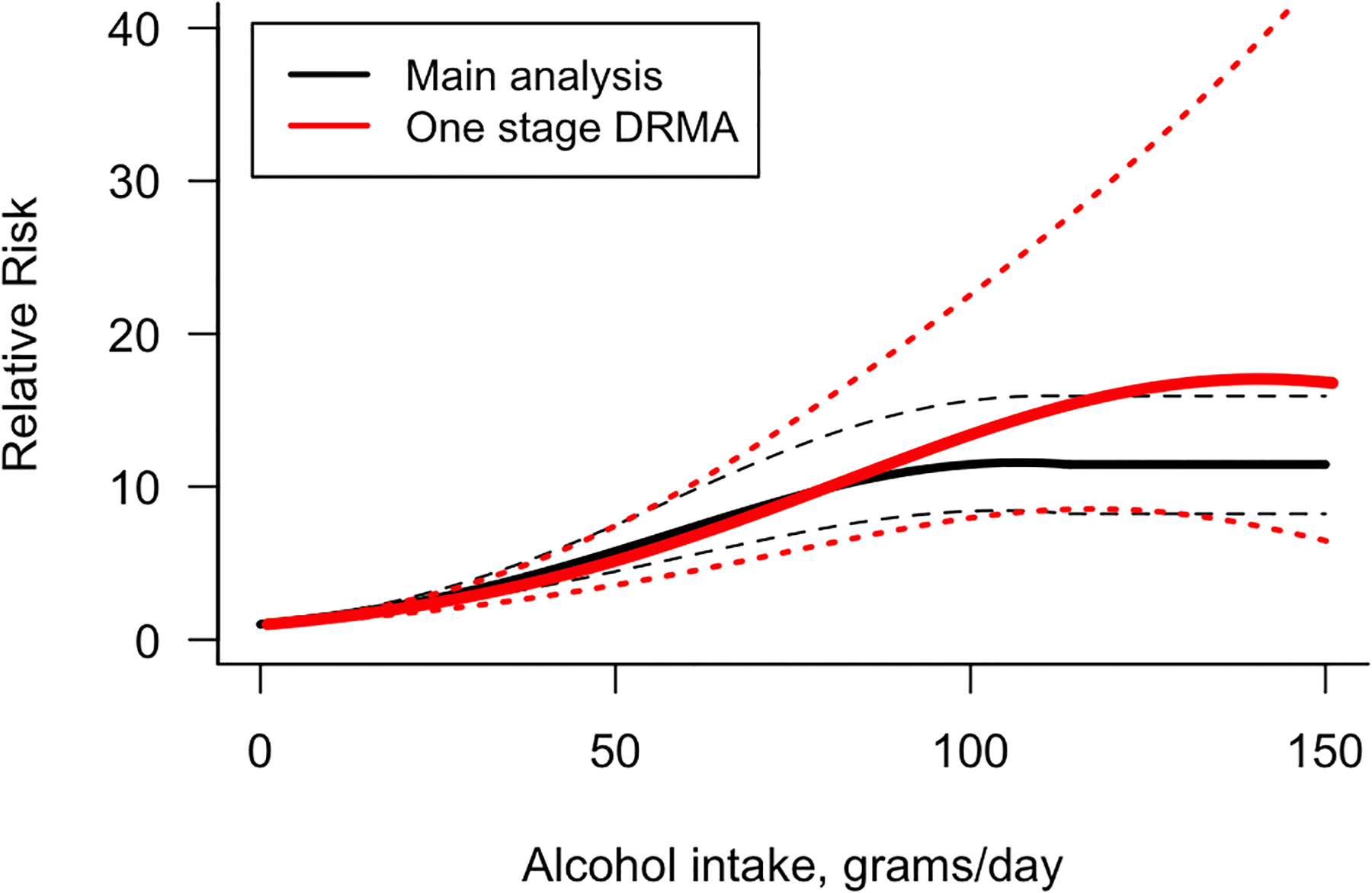
One-Stage Dose-Response Relationship Between Alcohol Consumption and All Cause Chronic Liver Disease Compared to Our Main Analysis

In the second sensitivity analysis, a quadratic dose-response relationship was identified. This one-stage dose-response meta-analysis provided higher estimates of the risk with wider confidence intervals, and the results using the proposed methodology included within those confidence intervals (Figure 3). Additionally, our proposed methods resulted in a better fitting model, as measured by AIC and BIC (AIC: 186.42 and BIC: 196.46), compared to the one-stage dose-response approach (AIC: 233.30 and BIC: 245.85). The most notable limitation of the one-stage dose-response is that only studies providing information on the number of cases per category can be included. As mentioned above, this meant that we had to exclude more than half of the papers identified in the systematic review. Therefore, the methodology proposed here provides a more precise alternative with a larger sample size when compared to the one-stage dose-response method.

## Conclusion

In summary, this paper proposes a three-step process for estimating dose-response relationships via meta-analysis and provides a practical example using R software. It provides detailed guidance on how data should be collected and analyzed with an approach that can improve the accuracy of modeling a dose-response curve based on a meta-analysis of studies. Notably, we intentionally omitted details of statistical theory in order to focus on the practical steps to implement these analyses, so that researchers across multiple disciplines can use these advanced statistical methods for not only alcohol use but other behavioral risk factors that may present a continuous, dose-response curve in the field of epidemiology as well.

One limitation of this approach is the challenge posed by studies with multiple risk factors, as they differ in how these factors are accounted for. Such variability can complicate both the generation and interpretation of a dose-response relationship for alcohol use. Future work could explore methods to better address these complexities and refine the proposed process further.

## Supplementary Material

Supplementary Materials

Dataset

For this article, the following Supplementary Materials are available:

- Code. (Jiang et al., 2025a)
- Dataset. (Jiang et al., 2025b)
- Study materials. (Jiang et al., 2025a)

## Figures and Tables

**Table 1:** Example of Four Papers (Risk Estimates as Given) and the Data Requirements

Paper	Dose (mean grams per day)	RR (95% CI)	SE	Control Variables
Åberg et al., 2020	Lifetime abstainers	Reference	n/a	Age, sex, BMI, smoking, exercise, diabetes, employment, marital status and education
Åberg et al. 2020	4.5	1.37 (0.72–2.59)	0.33	
Åberg et al. 2020	14.5	2.24 (1.06–4.75)	0.38	
Åberg et al. 2020	24.5	3.83 (1.76–8.35)	0.40	
Åberg et al. 2020	34.5	4.61 (2.04–10.43)	0.42	
Åberg et al. 2020	44.5	9.34 (4.14–21.09)	0.42	
Ioannou et al., 2005	Current abstainers	Reference	n/a	Age, BMI, geographical region, gender, race, household income, and educational attainment
Ioannou et al., 2005	7	1.51 (0.90–2.50)	0.26	
Ioannou et al., 2005	21	2.51 (1.20–5.40)	0.38	
Ioannou et al., 2005	38.5	5.14 (2.60–10.00)	0.34	
Persson et al., 2013	Current abstainers	1.88 (1.55–2.28)	0.10	Sex, age, race, education, smoking, BMI, and diabetes.
Persson et al., 2013	7	Reference	n/a	
Persson et al., 2013	28	2.03 (1.63–2.52)	0.11	
Persson et al., 2013	63	5.84 (4.81–7.10)	0.10	
Schwartz et al., 2013	2.7	Reference	n/a	Age, education, smoking, BMI, diabetes and study arm
Schwartz et al., 2013	12.9	2.06 (1.22–3.50)	0.27	
Schwartz et al., 2013	31.8	6.68 (4.16–10.71)	0.24	

Note: The SE can be derived from the 95% confidence intervals usually given in epidemiological studies.

**Table 2:** Example of Four Papers Rescaled to Lifetime Abstention

Paper	Dose (mean grams per day)	RR (95% CI)	SE
Åberg et al., 2020	0	Reference	n/a
Åberg et al., 2020	4.5	1.37 (0.72–2.59)	0.33
Åberg et al., 2020	14.5	2.24 (1.06–4.75)	0.38
Åberg et al., 2020	24.5	3.83 (1.76–8.35)	0.40
Åberg et al., 2020	34.5	4.61 (2.04–10.43)	0.42
Åberg et al., 2020	44.5	9.34 (4.14–21.09)	0.42
Ioannou et al., 2005	0	Reference	n/a
Ioannou et al., 2005	7	2.22 (1.32–3.67)	0.26
Ioannou et al., 2005	21	3.69 (1.76–7.93)	0.38
Ioannou et al., 2005	38.5	7.55 (3.82–14.68)	0.34
Persson et al., 2013	0	Reference	n/a
Persson et al., 2013	7	0.78 (0.63–0.97)	0.11
Persson et al., 2013	28	1.59 (1.27–1.97)	0.11
Persson et al., 2013	63	4.56 (3.76–5.55)	0.10
Schwartz et al., 2013	0	Reference	n/a
Schwartz et al., 2013	2.7	1.07 (1.06–1.08)	0.004
Schwartz et al., 2013	12.9	2.20 (1.30–3.74)	0.27
Schwartz et al., 2013	31.8	7.14 (4.45–11.45)	0.24

Note: The SE can be derived from the calculated 95% confidence intervals.

**Table 3:** Dose-Response Relationship Between Alcohol Consumption and Chronic Liver Disease by Sex for All Cause of Chronic Liver Disease.

${\text{RR}}_{\text{LC}\_\text{women}}$	${\text{RR}}_{\text{LC}\_\text{men}}$
$=\left\{\begin{array}{c} \text{exp}\left(\beta_{0}+\beta_{1}x+\beta_{2}x^{2}\right)\;x<82.5\\ \text{exp}\left(\beta_{0}+\beta_{1}\times 74+\beta_{2}{\times 74}^{2}\right)\;x\geq 82.5 \end{array}\right.$	$=\left\{\begin{array}{c} \text{exp}\left(\beta_{0}+\beta_{1}x+\beta_{2}x^{2}\right)\;x<97.5\\ \text{exp}\left(\beta_{0}+\beta_{1}\times 79+\beta_{2}{\times 79}^{2}\right)\;x\geq 97.5 \end{array}\right.$
where	where
$\beta_{0}$ = −0.188095	$\beta_{0}$ = −0.188095
$\beta_{1}$ = 0.057945	$\beta_{1}$ = 0.039402
$\beta_{2}$ = −0.000354	$\beta_{2}$ = −0.000203
$\text{var}\left(\beta_{0}\right)=0.113535^{2}$	$\text{var}\left(\beta_{0}\right)=0.113535^{2}$
$\text{var}\left(\beta_{1}\right)=0.010636^{2}$	$\text{var}\left(\beta_{1}\right)=0.013051^{2}$
$\text{var}\left(\beta_{2}\right)=0.000085^{2}$	$\text{var}\left(\beta_{2}\right)=0.000107^{2}$

## Data Availability

The supplementary guide to conducting dose-response meta-analyses in epidemiology is available at Jiang et al. (2025a) and the liver disease dataset is available at Jiang et al. (2025b)

## Table of Abbreviations:

AIC: Akaike information criterion
BIC: Bayesian information criterion
HR: Hazards ratios
OR: Odds ratios
PRISMA: Preferred Reporting Items for Systematic Reviews and Meta-Analyses
REML: Restricted Maximum Likelihood
RR: Relative risks
LC: Liver cirrhosis
