# Supplementary material for: A Practical Guide to Conducting Dose-Response Meta-Analyses in Epidemiology
Source: Methodology (Gott). Author manuscript; Available in PMC 2026 Jan 8. (PMC12779110; doi:10.5964/meth.14733)
Supplement: Supplementary Materials [file NIHMS2100208-supplement-Supplementary_Materials.pdf]

**A Practical Guide to Conducting Dose-Response Meta-Analyses in Epidemiology**

Authors: Huan Jiang, Jürgen Rehm, Charlotte Probst, Alexander Tran, Shannon Lange, Laura Llamosas-Falcón

**Supplementary Materials**

**Table of contents**

**Supplementary Material A. Population, exposure, comparator, outcome, and study design (PECOS) criteria for the systematic review** .... 2

**Supplementary Material B. Search strategy** .... 3

**Supplementary Material C. Code example** .... 4

**Supplementary Material D. Summary of the three-step process** .... 6

**Supplementary Material E. Selection of studies** .... 7

**Supplementary Material F. Studies included in the analysis** .... 8

**References** .... 14

**Supplementary Material A. Population, exposure, comparator, outcome, and study design (PECOS) criteria for the systematic review**

| | Eligibility criteria |
|---|---|
| Population | Adult population (i.e. 18 years or older) with no previous diagnosis of chronic liver disease |
| Exposure | Quantity of alcohol use |
| Comparator | Lifetime abstainers, current abstainers (including both former drinkers and lifetime abstainers), or the lowest alcohol consumption category. |
| Outcome | Chronic liver disease morbidity (incidence or decompensated liver cirrhosis) and mortality (defined by ICD-8 and ICD-9 codes 571 and ICD-10 codes K70-K76). |
| Study design | Case-control or cohort study design. We included studies that reported odds ratios, relative risks, or hazard ratios and their 95% confidence intervals, or information allowing us to compute them |

**Supplementary Material B. Search strategy**

We conducted a systematic review on the relationship between alcohol consumption and chronic liver disease using the databases PubMed/Medline and Embase from inception to October 12, 2021 applying the Preferred Reporting Items for Systematic Reviews and Meta-Analyses (PRISMA) criteria. The inclusion criteria were as presented in our PECO table (Supplementary Material Table e1). We excluded studies that were not published as full reports and studies with not enough data to compute the risk. All references were screened by one author, with independent verification by two additional reviewers. Full-text screening and data extraction was performed by two authors.

**PubMed/Medline**

("Case-Control Studies"[Mesh] OR "Cohort Studies"[Mesh] OR "Follow-Up Studies"[Mesh] OR "Longitudinal Studies"[Mesh]) AND (alcohol OR alcohol consumption OR "Alcohol Drinking"[Mesh] OR alcohol use disorders OR alcohol dependence OR binge drinking OR heavy drinking OR "alcohol-related disorders"[MeSH] OR alcoholism OR intoxicat* OR drunk*) AND liver cirrhosis AND (liver disease OR cirrhosis OR "Liver Cirrhosis"[Mesh] OR "Liver Cirrhosis/mortality"[Mesh]) AND ("Liver Diseases, Alcoholic"[Mesh] OR "Liver Cirrhosis, Alcoholic"[Mesh] OR hepatitis B OR HBV OR "Hepatitis B, Chronic"[Mesh] OR hepatitis C OR HCV OR "Hepatitis C, Chronic"[Mesh] OR metabolic disease OR "Metabolic Syndrome"[Mesh] OR "Non-alcoholic Fatty Liver Disease"[Mesh] OR "Hepatitis, Autoimmune"[Mesh])

**EMBASE**

| # ▲ | Searches |
|---|---|
| | **Study types** |
| 1 | exp Case-Control Studies/ |
| 2 | exp cohort studies/ or exp follow-up studies/ or exp longitudinal studies/ |
| 3 | 1 or 2 |
| | **Alcohol terms** |
| 4 | exp alcohol/exp and alcohol:kw,ab or exp alcohol consumption/ or exp alcohol drinking/ |
| 5 | 'alcohol dependence' or 'binge drinking' or exp heavy drinking/ or exp alcohol-related disorders/ or 'alcoholism' or exp alcoholism |
| 6 | intoxicat* or drunk* |
| 7 | 4 or 5 or 6 |
| | **Disease terms** |
| 8 | 'liver cirrhosis' or exp cirrhosis/ or exp liver cirrhosis, mortality/ or liver disease:kw,ab |
| | **Specifics** |
| 9 | exp liver cirrhosis, alcoholic/ |
| 10 | exp hepatitis B/exp or 'HBV' or exp hepatitis B, chronic/exp |
| 11 | exp hepatitis C/ or 'HCV' or exp hepatitis C, chronic/ |
| 12 | exp metabolic disease/ or exp metabolic syndrome/ or exp non-alcoholic fatty liver disease/ |
| 13 | exp autoimmune hepatitis/ |
| 14 | 9 or 10 or 11 or 12 or 13 |
| 15 | 3 and 7 and 8 and 14 |
| 16 | remove duplicates from 15 |

**Supplementary Material C. Code example**

```
library(tidyverse)
library(mvmeta)
library(meta)
library(metafor)
library(rms)
library(readxl)

#read the dataset
library(readxl)
#insert in the following brackets the folder where the dataset is located
final <- read_excel("Documents/workplace/liverdiseasedataset_forpublication.xlsx")

final <- final %>%
  filter(dose != 0.00)

factor_columns <- c("sex", "type", "usa", "qualitySC", "mortality")
final[factor_columns] <- lapply(final[factor_columns], as.factor)

###FIRST STEP: MODEL SELECTION

##LINEAR REGRESSION
linear <- rma.mv(yi=lnor, V=se^2, mods = ~ dose+0, data=final, random = list(~ 1 | lineid, ~ 1 |
study), method = "REML")
summary(linear)

##QUADRATIC REGRESSION
quad <- rma.mv(yi=lnor, V=se^2, mods = ~ dose + I(dose^2)+0, data=final, random = list(~ 1 |
lineid, ~ 1 | study), method = "REML")
summary(quad)

##RESTRICTED CUBIC SPLINE
s <- seq(0,150,length=150)
knots <- quantile(final$dose, c(.05, .35, .65, .95))
rcs <- rma.mv(yi= lnor ~ rcs(dose, knots)+0, V=se^2, data=final, random = list(~ 1 | lineid, ~ 1 |
study), method = "REML")
summary(rcs)

##CUBIC POLYNOMIAL MODEL
cp <- rma.mv(yi= lnor, V=se^2, mods = ~ poly(dose, degree=3, raw=TRUE)+0, data=final,
random = list(~ 1 | lineid, ~ 1 | study), method = "REML")

##MODEL COMPARISON
fitstats(linear, quad, rcs, cp)

###SECOND STEP: Inclusion of variables

model1 <- rma.mv(yi=lnor, V=se^2, mods = ~ dose+ I(dose^2) + dose*sex, data=final, random =
list(~ 1 | lineid, ~ 1 | study), method = "REML")
```

```
model1a <- rma.mv(yi=lnor, V=se^2, mods = ~ dose+ I(dose^2) + dose:sex, data=final, random
= list(~ 1 | lineid, ~ 1 | study), method = "REML")

model2 <- rma.mv(yi=lnor, V=se^2, mods = ~ dose+ I(dose^2) + dose:sex + I(dose^2):sex,
data=final, random = list(~ 1 | lineid, ~ 1 | study), method = "REML")

model3 <- rma.mv(yi=lnor, V=se^2, mods = ~ dose+ I(dose^2) + dose:sex + I(dose^2):sex +
dose*mortality, data=final, random = list(~ 1 | lineid, ~ 1 | study), method = "REML")

model3a <- rma.mv(yi=lnor, V=se^2, mods = ~ dose+ I(dose^2) + dose:sex + I(dose^2):sex +
dose:mortality, data=final, random = list(~ 1 | lineid, ~ 1 | study), method = "REML")

model4 <- rma.mv(yi=lnor, V=se^2, mods = ~ dose+ I(dose^2) + dose:sex+ I(dose^2):sex +
dose:mortality + I(dose^2):mortality, digits = 6, data=final, random = list(~ 1 | lineid, ~ 1 |
study), method = "REML")

#final model
model5 <- rma.mv(yi=lnor, V=se^2, mods = ~ dose+ I(dose^2) + dose:sex+ I(dose^2):sex +
dose:type + I(dose^2):type + dose:qualitySC + dose:usa + dose:mortality + I(dose^2):mortality,
digits = 6,data=final, random = list(~ 1 | lineid, ~ 1 | study), method = "REML")

# MODEL COMPARISON
AIC(model1, model1a, model2, model3, model3a, model4, model5)
BIC(model1, model1a, model2, model3, model3a, model4, model5)

# CHECK VARIANCE-COVARIANCE MATRIX
vcov(model5, type="fixed")
```

**Supplementary Material D. Summary of the three-step process**

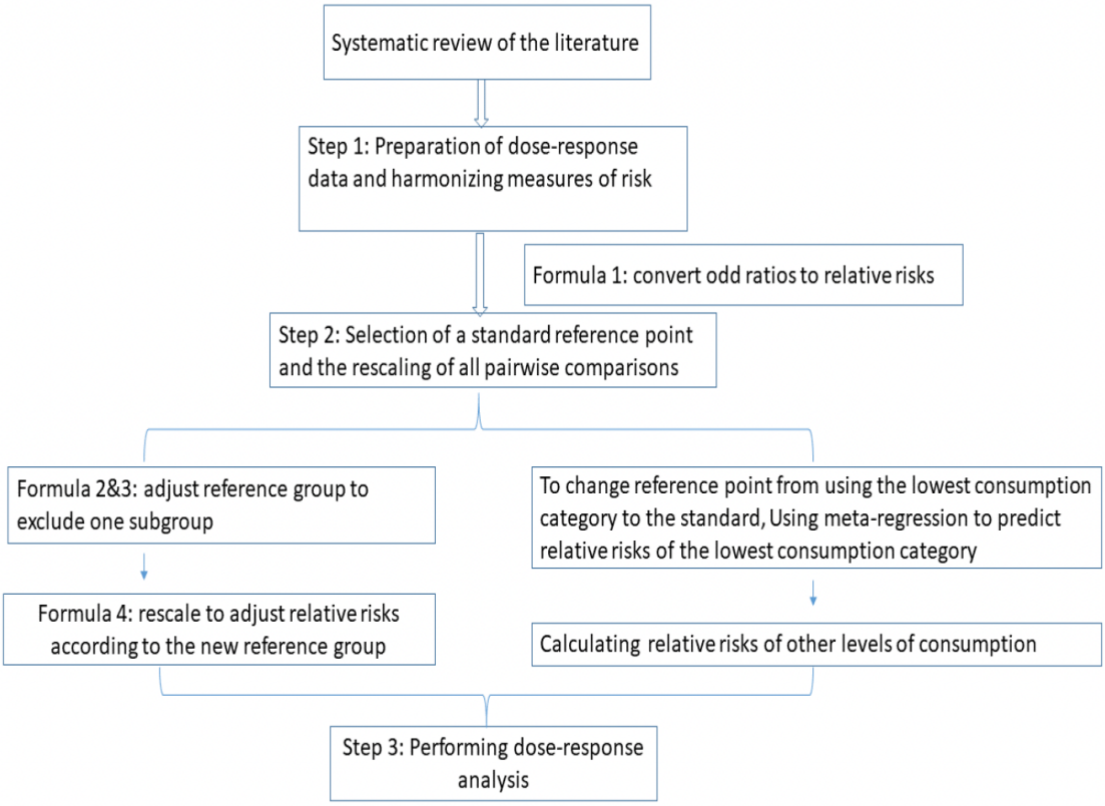

**Supplementary Material E. Selection of studies**

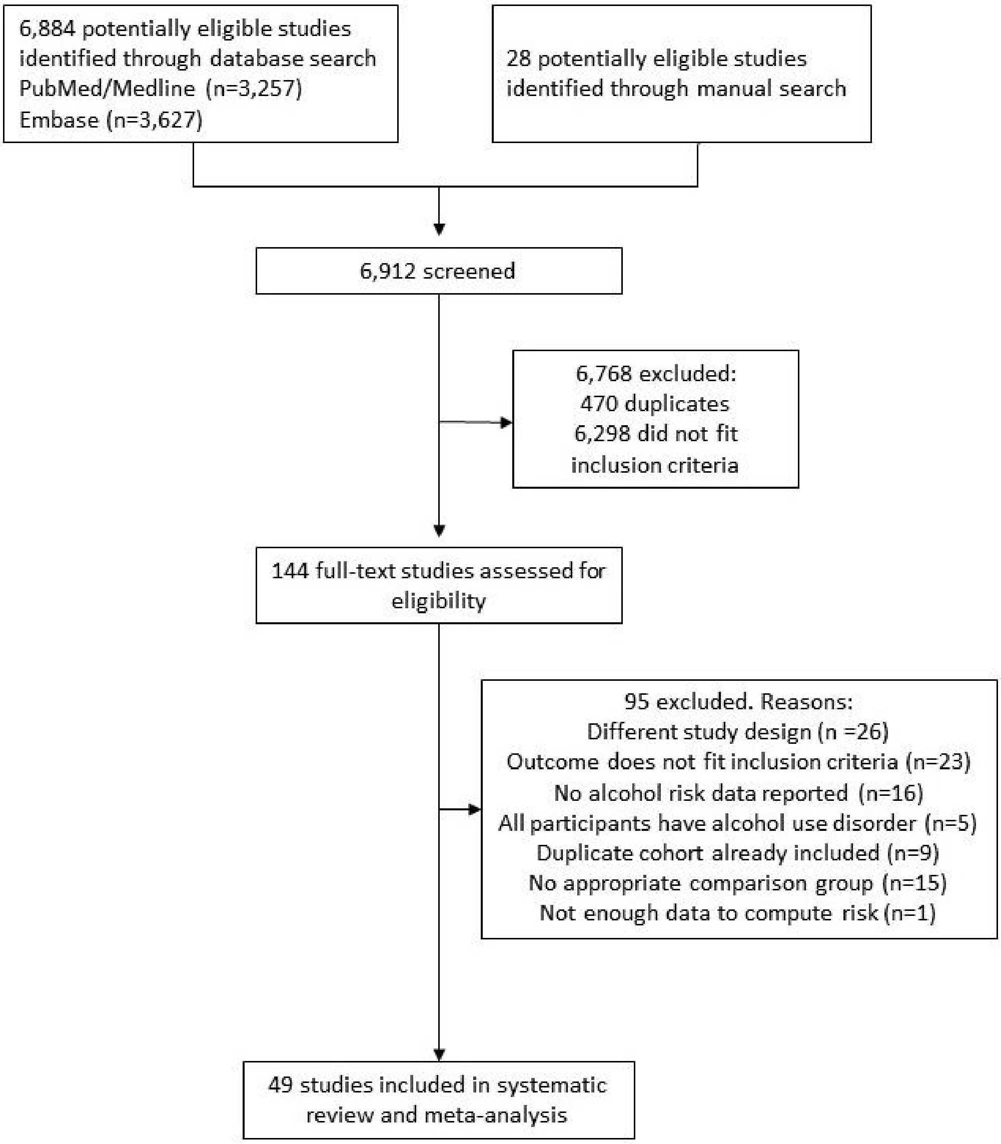

**Supplementary Material F. Studies included in the analysis**

| Study, year, country | N total | LD type | Alcohol use in g/day: n | Risk estimates | CI 95% lower | CI 95% upper | Outcome* N cases |
|---|---|---|---|---|---|---|---|
| Aberg et al.<br>2019[1]<br>Finland | 8,345 | Metabolic | Both sexes combined<br>LTA: 993<br>0-9 g/d: 4,429<br>10-19 g/d: 1,448<br>20-29 g/d: 761<br>30-39 g/d: 460<br>40-49 g/d: 254 | <br>Reference<br>1.37<br>2.24<br>3.83<br>4.61<br>9.34 | <br>-<br>0.72<br>1.06<br>1.76<br>2.04<br>4.14 | <br>-<br>2.59<br>4.75<br>8.35<br>10.43<br>21.09 | Mortality<br>13<br>59<br>25<br>22<br>16<br>17 |
| Alemy-Carreau<br>et al. 1996[2]<br>France | 221 | Other | Men<br>0 g/d: 24<br>100 g/d: 197 | <br>Reference<br>121.1 | <br>-<br>49.4 | <br>-<br>298.9 | Morbidity: 101 |
| Askgaard et al.<br>2015[3]<br>Denmark | 55,917 | ALD | Men<br>0-24 g/d: 15,028<br>24-48 g/d: 6,800<br>48-72 g/d: 2,774<br>72-96 g/d: 1,062<br>96-120 g/d: 445<br>>120 g/d: 174 | <br>Reference<br>2.33<br>6.98<br>13.12<br>29.03<br>50 | <br>-<br>1.52<br>4.65<br>8.51<br>18.63<br>30.12 | <br>-<br>3.58<br>10.5<br>20.23<br>45.24<br>82.96 | Mortality: 257 |
| | | | Women<br>0-24 g/d: 23,278<br>24-48 g/d: 4,242<br>48-72 g/d: 744<br>>72 g/d: 222 | <br>Reference<br>3.49<br>16.20<br>21.57 | <br>-<br>2.00<br>9.16<br>10.38 | <br>-<br>6.12<br>28.70<br>44.84 | Mortality: 85 |
| Batey et al.<br>1992[4]<br>Australia | 158 | All type | Men<br>≤40 g/d: 109<br>41-80 g/d:23<br>>80 g/d: 26 | <br>Reference<br>8.80<br>21.90 | <br>-<br>3.20<br>7.70 | <br>-<br>24.30<br>62.90 | Morbidity: 43 |
| Becker et al.<br>2002[5]<br>Denmark | 30,630 | ALD | Men<br><1.7 g/d<br>1.7-12 g/d<br>12-36 g/d<br>36-60 g/d<br>>60 g/d | <br>7.76<br>Reference<br>2.34<br>1.34<br>2.63 | <br>3.35<br>-<br>1.18<br>0.70<br>1.39 | <br>18.00<br>-<br>4.62<br>2.56<br>5 | Mortality: 212 |
| | | | Women<br><1.7 g/d<br>1.7-12 g/d<br>12-36 g/d<br>36-60 g/d<br>>60 g/d | <br>1.11<br>Reference<br>4.48<br>9.08<br>11.85 | <br>0.43<br>-<br>2.21<br>3.60<br>3.74 | <br>2.84<br>-<br>9.08<br>22.77<br>37.48 | Mortality: 80 |
| Bedogni et al.<br>2008[6]<br>Italy | 139 | HCV | Both sexes combined<br>0 g/d<br>30 g/d<br>60 g/d | <br>Reference<br>4.15<br>17.22 | <br>-<br>1.02<br>4.23 | <br>-<br>16.88<br>170.98 | Morbidity: 17 |
| Bellentani et al.<br>1997[7]<br>Italy | 6442 | All type | Both sexes combined<br>0-30 g/d: 5,167<br>31-60 g/d: 745<br>61-90 g/d: 276<br>91-120 g/d: 132<br>>120 g/d: 122 | <br>Reference<br>10.90<br>25.00<br>52.90<br>62.30 | <br>-<br>3.60<br>7.90<br>16.60<br>20.10 | <br>-<br>33.50<br>79.30<br>169.00<br>193.00 | Morbidity: 35 |
| Blackwelder et<br>al. 1980[8]<br>USA | 7888 | All type | Men<br>0 g/d: 3,747<br>1-10 g/d: 1,316<br>11-30 g/d: 1,593<br>≥30 g/d: 1,232 | <br>2.11<br>Reference<br>1.65<br>7.48 | <br>0.25<br>-<br>0.15<br>0.92 | <br>17.49<br>-<br>18.20<br>60.69 | Mortality<br>4<br>1<br>2<br>7 |

| Study, year, country | N total | LD type | Alcohol use in g/day: n | Risk estimates | CI 95% lower | CI 95% upper | Outcome* N cases |
|---|---|---|---|---|---|---|---|
| Boffetta et al. 1990[9] USA | 276,802 | All type | Men | | | | Mortality |
| | | | 0 g/d: 153,043 | Reference | - | - | 107 |
| | | | 14 g/d: 33,229 | 1.21 | 0.86 | 1.69 | 44 |
| | | | 28 g/d: 23,558 | 3.15 | 2.39 | 4.16 | 82 |
| | | | 42 g/d: 11,257 | 5.39 | 4 | 7.26 | 67 |
| | | | 56 g/d: 7,309 | 8.67 | 6.45 | 11.6 | 71 |
| | | | 70 g/d: 3,368 | 10.6 | 7.36 | 15.3 | 39 |
| | | | ≥84 g/d: 7,698 | 18.1 | 14.1 | 23.2 | 153 |
| Corrao et al. 1993[10] Italy | 640 | All type | Men | | | | Morbidity |
| | | | LTA: 28 | Reference | - | - | 9 |
| | | | 25 or 50 g/d: 58 | 1.4 | 0.6 | 3.1 | 23 |
| | | | 75 or 100 g/d: 98 | 1.6 | 0.7 | 3.8 | 37 |
| | | | 125 or 150 g/d: 95 | 2.1 | 0.9 | 4.7 | 44 |
| | | | Women | | | | Morbidity |
| | | | LTA: 62 | Reference | - | - | 31 |
| | | | 25 or 50 g/d: 82 | 0.5 | 0.2 | 0.8 | 27 |
| | | | 75 or 100 g/d: 38 | 1.4 | 0.6 | 3.2 | 21 |
| | | | 125 or 150 g/d: 26 | 2.6 | 1 | 6.5 | 18 |
| Corrao et al. 1997[11] Italy | 1,113 | All type | Men | | | | Morbidity |
| | | | LTA: 48 | Reference | - | - | 6 |
| | | | <50 g/d: 255 | 1.9 | 0.62 | 5.8 | 51 |
| | | | 50-100 g/d: 132 | 9.1 | 2.94 | 28.18 | 70 |
| | | | ≥100 g/d: 220 | 31.4 | 10.3 | 95.76 | 173 |
| | | | Women | | | | Morbidity |
| | | | LTA: 104 | Reference | - | - | 24 |
| | | | <50 g/d: 306 | 2.09 | 0.69 | 6.3 | 101 |
| | | | 50-100 g/d: 35 | 7.5 | 3.46 | 16.25 | 27 |
| | | | ≥100 g/d: 13 | 20.36 | 6.3 | 65.79 | 10 |
| Di Martino et al. 2001[12] France | 160 | HCV | Both sexes combined | | | | Morbidity: 20 |
| | | | <80 g/d: 126 | Reference | - | - | |
| | | | >80 g/d: 34 | 2.65 | 0.62 | 11.32 | |
| Di Martino et al. 2011[13] France | 1107 | HCV | Both sexes combined | | | | Morbidity: 53 |
| | | | <50 g/d: 605 | Reference | - | - | |
| | | | >50 g/d: 352 | 4.29 | 2.26 | 8.13 | |
| Fuchs et al. 1995[14] USA | 85,709 | All type | Women | | | | Mortality |
| | | | 0 g/d: 25535 | 4.76 | 1.66 | 39.29 | 8 |
| | | | 0.1-1.4 g/d: 11304 | Reference | - | - | 1 |
| | | | 1.5-4.9 g/d: 18460 | 3.29 | 1.14 | 9.43 | 5 |
| | | | 5-14.9 g/d: 17783 | 6.05 | 2.57 | 14.33 | 10 |
| | | | 15-29.9 g/d: 8106 | 8.86 | 3.62 | 21.86 | 9 |
| | | | ≥30 g/d: 4521 | 12.14 | 5.05 | 29.10 | 15 |
| Garfinkel et al. 1988[15] USA | 581,321 | All type | Women | | | | Mortality |
| | | | LTA: 467,382 | Reference | - | - | 320 |
| | | | 14 g/d: 20,000 | 2.46 | 1.87 | 3.25 | 50 |
| | | | 28 g/d:13,000 | 7.40 | 5.90 | 9.27 | 75 |
| | | | 49 g/d: 10,000 | 14.21 | 10.77 | 18.75 | 50 |
| | | | 77 g/d: 12,000 | 16.70 | 12.10 | 23.04 | 39 |
| | | | ≥84 g/d: 2,000 | 28.29 | 20.21 | 39.59 | 34 |
| Goh et al. 2014[16] Singapore | 63,143 | All type | Both sexes combined | | | | Mortality |
| | | | 0 g/d: 51,384 | Reference | - | - | 58 |
| | | | <20 g/d: 10,278 | 0.72 | 0.39 | 1.35 | 12 |
| | | | ≥20 g/d: 1,595 | 7.07 | 4.01 | 12.47 | 18 |
| Gordon et al. 1984[17] | 4747 | All type | Men | | | | Mortality |
| | | | 0 g/d: 402 | Reference | - | - | 1 |

| **Study, year, country** | **N total** | **LD type** | **Alcohol use in g/day: n** | **Risk estimates** | **CI 95% lower** | **CI 95% upper** | **Outcome* N cases** |
|---|---|---|---|---|---|---|---|
| USA | | | 0.7-20 g/d: 1107<br>21-41 g/d: 344<br>41-61 g/d: 131<br>62-82 g/d: 50 | 1.45<br>3.51<br>9.21<br>8.04 | 0.16<br>0.37<br>0.97<br>0.51 | 12.96<br>33.55<br>87.75<br>126.55 | 4<br>3<br>3<br>1 |
| | | | Women<br>0-7-20 g/d: 2434<br>21-61 g/d: 191 | <br>Reference<br>1.59 | <br>-<br>0.2 | <br>-<br>12.67 | Mortality: NA |
| Gordon et al. 1987[18]<br>USA | 1,762 | All type | Men<br>0 g/d: 585<br>0.7-20 g/d: 842<br>21-41 g/d: 175<br>41-61 g//d: 100<br>> 62 g/d: 60 | <br>Reference<br>0.52<br>2.51<br>2.93<br>7.31 | <br>-<br>0.12<br>0.57<br>0.54<br>1.68 | <br>-<br>2.32<br>11.1<br>15.76<br>31.91 | Mortality<br>3<br>3<br>3<br>2<br>3 |
| Harris et al. 2002[19]<br>UK | 755 | HCV | Both sexes combined<br>0 g/d: 344<br><23 g/d: 224<br>≥23 g/d: 102 | <br>Reference<br>0.51<br>1.44 | <br>-<br>0.20<br>0.55 | <br>-<br>1.30<br>3.76 | Morbidity: 34 |
| Hart et al. 2010[20]<br>UK | 9,559 | All type | Men<br>0 g/d: 3,355<br>1-7 g/d: 1,817<br>8-14 g/d: 1,766<br>15-21 g//d: 1,017<br>22-34 g/d: 942<br>≥ 35 g/d: 662 | <br>Reference<br>1.50<br>1.24<br>5.17<br>6.81<br>10.50 | <br>-<br>0.60<br>0.47<br>2.32<br>3.10<br>4.74 | <br>-<br>3.70<br>3.27<br>11.50<br>14.90<br>23.30 | Mortality<br>7<br>9<br>7<br>16<br>19<br>19 |
| Im et al. 2021[21]<br>China | 218,341 | All type | Men<br>1 g/d: 117,072<br><20 g/d: 24,171<br>20-40 g/d: 18,182<br>40-60 g/d: 12,318<br>≥ 60 g/d: 12,318 | <br>Reference<br>1.29<br>2.47<br>3.13<br>8.15 | <br>-<br>1.06<br>2.10<br>2.59<br>7.04 | <br>-<br>1.57<br>2.91<br>3.79<br>9.43 | Morbidity: 1210 |
| | | | Women<br>1 g/d: 28,396<br>16 g/d: 5,896 | <br>Reference<br>1.09 | <br>-<br>0.71 | <br>-<br>1.68 | Morbidity: 946 |
| Innes et al. 2021[22]<br>UK | 489,285 | All type | Both sexes combined<br>0-12 g/d:374,142<br>25-38 g/d: 96,388<br>57-80 g/d: 18,755 | <br>Reference<br>1.25<br>4.91 | <br>-<br>1.12<br>4.35 | <br>-<br>1.39<br>5.54 | Morbidity: 2070 |
| Ioannou et al. 2005[23]<br>USA | 11,434 | All type | Both sexes combined<br>0 g/d: 4,744<br>0-14 g/d: 5,190<br>14-28 g/d: 848<br>>28 g/d: 652 | <br>Reference<br>1.51<br>2.51<br>5.14 | <br>-<br>0.90<br>1.20<br>2.60 | <br>-<br>2.50<br>5.40<br>10.00 | Mortality<br>18<br>35<br>10<br>17 |
| Khan et al. 2000[24]<br>Japan | 106 | HCV | Men<br>0 g/d: 40<br><80 g/d: 42<br>>80 g/d: 24 | <br>Reference<br>6.00<br>6.00 | <br>-<br>2.29<br>1.98 | <br>-<br>15.69<br>18.21 | Morbidity<br>7<br>28<br>16 |
| Khan et al. 1998[25]<br>Australia | 434 | HCV | Both sexes combined<br>0-80 g/d: 307<br>81-120 g/d: 69<br>>120 g/d: 35 | <br>Reference<br>0.78<br>1.22 | <br>-<br>0.39<br>0.53 | <br>-<br>1.58<br>2.82 | Morbidity: 79 |
| Klatsky et al. 1981[26]<br>USA | 8,060 | All type | Both sexes combined<br>0 g/d: 2,015<br><70 g/d: 4,030<br>>77 g/d: 2,015 | <br>Reference<br>1.20<br>6.60 | <br>-<br>0.42<br>2.58 | <br>-<br>2.40<br>16.87 | Mortality<br>3<br>12<br>33 |

| **Study, year, country** | **N total** | **LD type** | **Alcohol use in g/day: n** | **Risk estimates** | **CI 95% lower** | **CI 95% upper** | **Outcome* N cases** |
|---|---|---|---|---|---|---|---|
| Klatsky et al. | 128,934 | All type | Men | | | | Mortality |
| 2003[27] | | | LTA: 4,125 | Reference | - | - | 12 |
| USA | | | <0.5 g/d: 8,105 | 0.7 | 0.32 | 1.53 | 13 |
| | | | <14 g/d: 21,264 | 0.5 | 0.25 | 1.02 | 21 |
| | | | 14-28 g/d: 13,512 | 1.3 | 0.68 | 2.48 | 39 |
| | | | 42-70 g/d: 5,905 | 3.3 | 1.7 | 6.4 | 32 |
| | | | ≥84 g/d: 1,535 | 8.3 | 3.97 | 17.34 | 17 |
| | | | Women | | | | Mortality |
| | | | LTA: 11,373 | Reference | - | - | 7 |
| | | | <0.5 g/d: 19,312 | 1.2 | 0.42 | 3.42 | 8 |
| | | | <14 g/d: 26,631 | 2.5 | 1 | 6.26 | 13 |
| | | | 14-28 g/d: 9,896 | 4.7 | 2.02 | 10.95 | 20 |
| | | | 42-70 g/d: 2,523 | 14.2 | 5.94 | 33.96 | 18 |
| Kondili et al. | 301 | All type | Both sexes combined | | | | Morbidity: 106 |
| 1998[28] | | | 0-67 g/d: 269 | Reference | - | - | |
| Albania | | | 68-105 g/d: 11 | 3.6 | 0.6 | 21.4 | |
| | | | > 105 g/d: 21 | 8.6 | 1.8 | 40.8 | |
| Kono et al. | 5,135 | All type | Men | | | | Mortality |
| 1986[29] | | | LTA: 1,074 | Reference | - | - | 11 |
| Japan | | | <27 g/d: 1,034 | 0.30 | 0.10 | 1.10 | 3 |
| | | | >27 g/d: 925 | 1.80 | 0.80 | 4.00 | 14 |
| Liu et al. 2009[30] | 1290413 | All type | Women | | | | Morbidity |
| UK | | | 0 g/d: 305,652 | 1.41 | 1.23 | 1.61 | 381 |
| | | | 1-2 g/d:372,065 | Reference | - | - | 380 |
| | | | 3-7 g/d:294,353 | 1.24 | 1.08 | 1.44 | 363 |
| | | | 8-16 g/d: 241,307 | 1.84 | 1.60 | 2.11 | 466 |
| | | | ≥17 g/d: 67,360 | 4.32 | 3.71 | 5.03 | 322 |
| Monto et al. | 800 | HCV | Both sexes combined | | | | Morbidity: 84 |
| 2004[31] | | | 3.6 g/d: 200 | Reference | - | - | |
| USA | | | 3.6-17.3 g/d: 200 | 0.87 | 0.43 | 1.76 | |
| | | | 17.3–59 g/d: 200 | 1.06 | 0.52 | 2.08 | |
| | | | 59 g/d: 200 | 1.84 | 0.99 | 3.42 | |
| Norton et al. | 135 | All type | Women | | | | Morbidity: 36 |
| 1987[32] | | | <40 g/d: 102 | Reference | - | - | |
| Australia | | | >40 g/d: 33 | 784 | 84.52 | 7272.06 | |
| Patra et al. | 229,306 | All type | Both sexes combined | | | | Mortality |
| 2021[33] | | | LTA: 53455 | Reference | - | - | 37 |
| USA | | | <20 g/d: 125205 | 0.77 | 0.49 | 1.19 | 61 |
| | | | 20-40 g/d: 9125 | 2.55 | 1.41 | 4.63 | 20 |
| | | | >40-60 g/d: 3160 | 5.41 | 2.89 | 10.14 | 17 |
| | | | >60 g/d: 2005 | 10.25 | 5.64 | 18.62 | 22 |
| Pequignot et al. | 962 | All type | Men | | | | Morbidity: 184 |
| 1978[34] | | | 0-20 g/d: 188 | Reference | - | - | |
| France | | | 21-40 g/d: 222 | 3.1 | 0.84 | 11.42 | |
| | | | 41-60 g/d: 180 | 6.2 | 1.76 | 21.83 | |
| | | | 61-80 g/d: 132 | 13.8 | 4.06 | 46.92 | |
| | | | 81-100 g/d: 88 | 29.6 | 8.71 | 100.55 | |
| | | | 101-120 g/d: 54 | 41 | 11.61 | 144.83 | |
| | | | 121-140 g/d: 38 | 124.3 | 33.10 | 466.75 | |
| | | | >141 g/d: 60 | 659.3 | 159.57 | 2723.98 | |
| Persson et al. | 494,743 | All type | Both sexes combined | | | | Mortality |
| 2013[35] | | | 0 g/d: 118,926 | 1.88 | 1.55 | 2.28 | 144 |
| USA | | | <14 g/d: 260,769 | Reference | - | - | 225 |
| | | | 14-42 g/d: 75,821 | 2.03 | 1.63 | 2.52 | 135 |

| Study, year, country | N total | LD type | Alcohol use in g/day: n | Risk estimates | CI 95% lower | CI 95% upper | Outcome* N cases |
|---|---|---|---|---|---|---|---|
| | | | >42 g/d: 37,188 | 5.84 | 4.81 | 7.1 | 217 |
| Pol et al. 1998[36] France | 553 | HCV | Both sexes combined<br><80 g/d: 456<br>≥ 80 g/d: 97 | <br>Reference<br>2.90 | <br>-<br>1.60 | <br>-<br>5.40 | Morbidity: 69 |
| Pol et al. 1998[37] France | 210 | HCV | Both sexes combined<br><80 g/d: 134<br>≥80 g/d: 76 | <br>Reference<br>1.81 | <br>-<br>0.92 | <br>-<br>3.61 | Morbidity: 42 |
| Poynard et al. 2001[38] France | 2,313 | HCV | Both sexes combined<br><50 g/d: 2,119<br>≥50 g/d: 115 | <br>Reference<br>4.50 | <br>-<br>1.73 | <br>-<br>11.68 | Morbidity: 205 |
| Roudot-Thoraval et al. 1997[39] France | 5,789 | HCV | Both sexes combined<br><60 g/d: 4,515<br>>60 g/d: 1,042 | <br>Reference<br>3.38 | <br>-<br>2.82 | <br>-<br>4.05 | Morbidity: 1,237 |
| Schult et al. 2017[40] Sweden | 1,462 | All type | Women<br>0 g/d<br>10 g/d<br>30 g/d | <br>Reference<br>2.16<br>10.08 | <br>-<br>1.57<br>3.87 | <br>-<br>2.99<br>26.73 | Morbidity: 11 |
| Schwartz et al. 2013[41] Finland | 27,086 | All type | Men<br>0-5.33 g/d: 8,922<br>5.33-20.44 g/d: 9,119<br>>20.44 g/d: 9,045 | <br>Reference<br>2.06<br>6.68 | <br>-<br>1.22<br>4.16 | <br>-<br>3.50<br>10.71 | Mortality: 213 |
| Serfaty et al. 1997[42] France | 164 | HCV | Total<br><30 g/d: 113<br>30-80 g/d: 34<br>>80 g/d: 21 | <br>Reference<br>2.39<br>2.12 | <br>-<br>1.08<br>0.82 | <br>-<br>5.31<br>5.52 | Morbidity: 82 |
| Stroffolini et al. 2010[43] Italy | 397 | All type | Men<br>12-24 g/d: 215<br>36 g/d: 56<br>≥36 g/d: 239 | <br>Reference<br>1.2<br>4.3 | <br>-<br>0.5<br>2.5 | <br>-<br>2.9<br>7.3 | Morbidity: 111 |
| | | | Women<br>12-24 g/d: 204<br>36 g/d: 18<br>≥36 g/d: 37 | <br>Reference<br>0.8<br>5.7 | <br>-<br>0.1<br>2.3 | <br>-<br>6.5<br>14.5 | Morbidity: 26 |
| Trembling et al. 2017[44] UK | 95,126 | All type | Women<br>0 g/d: 22,215<br>1-15 g/d: 68,608<br>16-20 g/d: 2,881<br>≥21 g/d: 1,422 | <br>Reference<br>0.78<br>0.97<br>1.83 | <br>-<br>0.61<br>0.52<br>0.97 | <br>-<br>1.00<br>1.82<br>3.44 | Mortality<br>70<br>202<br>11<br>11 |
| Tverdal et al. 2018[45] Norway | 219,279 | ALD | Both sexes combined<br>0 g/d: 71,230<br><12 g/d: 98,612<br>12- 24 g/d: 35,268<br>≥ 24 g/d: 14,169 | <br>Reference<br>1.4<br>2.42<br>10.39 | <br>-<br>0.75<br>1.22<br>5.61 | <br>-<br>2.6<br>4.81<br>19.24 | Mortality<br>10<br>29<br>18<br>31 |
| Wiley et al. 2002[46] USA | 176 | HCV | Both sexes combined<br><40 g/d in W and <60 g/d in M: 86<br>>40 g/d in W and >60 g/d in M: 90 | <br>Reference<br>4.41 | <br>-<br>2.28 | <br>-<br>8.51 | Morbidity: 69 |
| Yang et al. 2012[47] China | 218,189 | All type | Men<br>0 g/d: 145,323<br><20 g/d: 14,208<br>20-40 g/d: 19,391<br>40-60 g/d: 18,681<br>60-100 g/d: 10,870 | <br>Reference<br>1.12<br>0.84<br>1.16<br>1.22 | <br>-<br>0.76<br>0.59<br>0.85<br>0.83 | <br>-<br>1.64<br>1.21<br>1.56<br>1.79 | Mortality<br>176<br>27<br>30<br>44<br>27 |

| Study, year, country | N total | LD type | Alcohol use in g/day: n | Risk estimates | CI 95% lower | CI 95% upper | Outcome* N cases |
|---|---|---|---|---|---|---|---|
| | | | ≥100 g/d: 9,716 | 1.74 | 1.22 | 2.48 | 35 |
| Yi et al. 2016[48] South Korea | 107,735 | All type | Men | | | | Mortality: 178 |
| | | | <1.3 g/d: 34,435 | Reference | - | - | |
| | | | 1.3-8 g/d: 31,341 | 1.32 | 0.81 | 2.18 | |
| | | | 9-17 g/d: 15,162 | 2.45 | 1.48 | 4.07 | |
| | | | 18-35 g/d: 15,764 | 2.77 | 1.71 | 4.50 | |
| | | | ≥36 g/d: 11,033 | 4.03 | 2.51 | 6.46 | |
| Yuan et al. 1997[49] China | 18,244 | All type | Men | | | | Mortality |
| | | | LTA: 10,471 | Reference | - | - | 23 |
| | | | 1.7-48 g/d: 6,189 | 0.46 | 0.17 | 1.22 | 6 |
| | | | ≥50 g/d: 1,201 | 2.99 | 1.12 | 7.94 | 6 |

Abbreviations: LD, liver disease; CI, confidence intervals; ALD, alcohol-related liver disease; HCV, hepatitis C virus, USA, United States of America; UK, United Kingdom
*Some studies only provided the total number of cases and not the number of cases per alcohol category

**References**

1. Åberg F, Puukka P, Salomaa V, Männistö S, Lundqvist A, Valsta L, et al. Risks of Light and Moderate Alcohol Use in Fatty Liver Disease: Follow-Up of Population Cohorts. Hepatology. 2020;71(3):835-48.
2. Alemy-Carreau M, Durbec J-P, Giordanella J, Rousseau S, Blanc G, Monges D, et al. Lack of interaction between hepatitis C virus and alcohol in the pathogenesis of cirrhosis. A statistical study. Journal of hepatology. 1996;25(5):627-32.
3. Askgaard G, Grønbæk M, Kjær MS, Tjønneland A, Tolstrup JS. Alcohol drinking pattern and risk of alcoholic liver cirrhosis: A prospective cohort study. Journal of Hepatology. 2015;62(5):1061-7.
4. Batey RG, Burns T, Benson RJ, Byth K. Alcohol consumption and the risk of cirrhosis. Medical Journal of Australia. 1992;156(6):413-6.
5. Becker U, Grønbæk M, Johansen D, Sørensen TI. Lower risk for alcohol-induced cirrhosis in wine drinkers. Hepatology. 2002;35(4):868-75.
6. Bedogni G, Miglioli L, Masutti F, Ferri S, Castiglione A, Lenzi M, et al. Natural course of chronic HCV and HBV infection and role of alcohol in the general population: the Dionysos Study. Official journal of the American College of Gastroenterology| ACG. 2008;103(9):2248-53.
7. Bellentani S, Saccoccio G, Costa G, Tiribelli C, Manenti F, Sodde M, et al. Drinking habits as cofactors of risk for alcohol induced liver damage. Gut. 1997;41(6):845-50.
8. Blackwelder WC, Yano K, Rhoads GG, Kagan A, Gordon T, Palesch Y. Alcohol and mortality: the Honolulu heart study. The American journal of medicine. 1980;68(2):164-9.
9. Boffetta P, Garfinkel L. Alcohol drinking and mortality among men enrolled in an American Cancer Society prospective study. Epidemiology. 1990:342-8.
10. Corrao G, Arico S, Lepore AR, Valenti M, Torchio P, Galatola G, et al. Amount and duration of alcohol intake as risk factors of symptomatic liver cirrhosis: a case—control study. Journal of clinical epidemiology. 1993;46(7):601-7.
11. Corrao G, Arico S, Zambon A, Torchio P, Orio FD. Female sex and the risk of liver cirrhosis. Scandinavian journal of gastroenterology. 1997;32(11):1174-80.
12. Di Martino V, Rufat P, Boyer N, Renard P, Matheron S, Le Moing V, et al. The influence of human immunodeficiency virus coinfection on chronic hepatitis C in injection drug users: a long-term retrospective cohort study. Hepatology. 2001;34(6):1193-9.
13. Di Martino V, Crouzet J, Hillon P, Thévenot T, Minello A, Monnet E. Long-term outcome of chronic hepatitis C in a population-based cohort and impact of antiviral therapy: a propensity-adjusted analysis. Journal of Viral Hepatitis. 2011;18(7):493-505.
14. Fuchs CS, Stampfer MJ, Colditz GA, Giovannucci EL, Manson JE, Kawachi I, et al. Alcohol consumption and mortality among women. New England Journal of Medicine. 1995;332(19):1245-50.
15. Garfinkel L, Boffetta P, Stellman SD. Alcohol and breast cancer: a cohort study. Preventive Medicine. 1988;17(6):686-93.
16. Goh GB-B, Chow W-C, Wang R, Yuan J-M, Koh W-P. Coffee, alcohol and other beverages in relation to cirrhosis mortality: The Singapore Chinese Health Study. Hepatology. 2014;60(2):661-9.
17. Gordon T, Kannel WB. Drinking and mortality: The Framingham study. American journal of epidemiology. 1984;120(1):97-107.
18. Gordon T, Doyle JT. Drinking and mortality: the Albany Study. American journal of epidemiology. 1987;125(2):263-70.
19. Harris HE, Ramsay ME, Andrews N, Eldridge KP. Clinical course of hepatitis C virus during the first decade of infection: cohort study. Bmj. 2002;324(7335):450.

20. Hart CL, Morrison DS, Batty GD, Mitchell RJ, Davey Smith G. Effect of body mass index and alcohol consumption on liver disease: analysis of data from two prospective cohort studies. Bmj. 2010;340:c1240.
21. Im PK, Millwood IY, Kartsonaki C, Guo Y, Chen Y, Turnbull I, et al. Alcohol drinking and risks of liver cancer and non-neoplastic chronic liver diseases in China: a 10-year prospective study of 0.5 million adults. BMC Medicine. 2021;19(1):216.
22. Innes H, Crooks CJ, Aspinall E, Card TR, Hamill V, Dillon J, et al. Characterizing the risk interplay between alcohol intake and body mass index on cirrhosis morbidity. Hepatology. 2021;n/a(n/a).
23. Ioannou GN, Weiss NS, Boyko EJ, Kowdley KV, Kahn SE, Carithers RL, et al. Is central obesity associated with cirrhosis-related death or hospitalization? A population-based, cohort study. Clin Gastroenterol Hepatol. 2005;3(1):67-74.
24. Khan KN, Yatsuhashi H. Effect of alcohol consumption on the progression of hepatitis C virus infection and risk of hepatocellular carcinoma in Japanese patients. Alcohol and Alcoholism. 2000;35(3):286-95.
25. Khan MH, Thomas L, Byth K, Kench J, Weltman M, George J, et al. How much does alcohol contribute to the variability of hepatic fibrosis in chronic hepatitis C? Journal of gastroenterology and hepatology. 1998;13(4):419-26.
26. Klatsky AL, Friedman GD, Siegelaub AB. Alcohol and mortality: a ten-year Kaiser-Permanente experience. Annals of Internal Medicine. 1981;95(2):139-45.
27. Klatsky AL, Friedman GD, Armstrong MA, Kipp H. Wine, liquor, beer, and mortality. American journal of epidemiology. 2003;158(6):585-95.
28. Kondili L, Tosti M, Szklo M, Costantino A, Cotichini R, Resuli B, et al. The relationships of chronic hepatitis and cirrhosis to alcohol intake, hepatitis B and C, and delta virus infection: a case-control study in Albania. Epidemiology & Infection. 1998;121(2):391-5.
29. Kono S, Ikeda M, Tokudome S, Nishizumi M, Kuratsune M. Alcohol and mortality: a cohort study of male Japanese physicians. International Journal of Epidemiology. 1986;15(4):527-32.
30. Liu B, Balkwill A, Roddam A, Brown A, Beral V. Separate and joint effects of alcohol and smoking on the risks of cirrhosis and gallbladder disease in middle-aged women. American journal of epidemiology. 2009;169(2):153-60.
31. Monto A, Patel K, Bostrom A, Pianko S, Pockros P, McHutchison JG, et al. Risks of a range of alcohol intake on hepatitis C-related fibrosis. Hepatology. 2004;39(3):826-34.
32. Norton R, Batey R, Dwyer T, MacMahon S. Alcohol consumption and the risk of alcohol related cirrhosis in women. Br Med J (Clin Res Ed). 1987;295(6590):80-2.
33. Patra J, Buckley C, Kerr WC, Brennan A, Purshouse RC, Rehm J. Impact of body mass and alcohol consumption on all-cause and liver mortality in 240 000 adults in the United States. Drug and alcohol review. 2021.
34. Pequignot G, Tuyns AJ, Berta JL. Ascitic cirrhosis in relation to alcohol consumption. Int J Epidemiol. 1978;7(2):113-20.
35. Persson EC, Schwartz LM, Park Y, Trabert B, Hollenbeck AR, Graubard BI, et al. Alcohol consumption, folate intake, hepatocellular carcinoma, and liver disease mortality. Cancer Epidemiology and Prevention Biomarkers. 2013;22(3):415-21.
36. Pol S, Fontaine H, Carnot F, Zylberberg H, Berthelot P, Bréchot C, et al. Predictive factors for development of cirrhosis in parenterally acquired chronic hepatitis C: a comparison between immunocompetent and immunocompromised patients. Journal of hepatology. 1998;29(1):12-9.
37. Pol S, Lamorthe B, Thi NT, Thiers V, Carnot F, Zylberberg H, et al. Retrospective analysis of the impact of HIV infection and alcohol use on chronic hepatitis C in a large cohort of drug users. Journal of hepatology. 1998;28(6):945-50.

38. Poynard T, Ratziu V, Charlotte F, Goodman Z, McHutchison J, Albrecht J. Rates and risk factors of liver fibrosis progression in patients with chronic hepatitis C. Journal of hepatology. 2001;34(5):730-9.
39. Roudot-Thoraval F, Bastie A, Pawlotsky J, Dhumeaux D. Epidemiological factors affecting the severity of hepatitis C virus-related liver disease: a French survey of 6,664 patients. Hepatology. 1997;26(2):485-90.
40. Schult A, Mehlig K, Björkelund C, Wallerstedt S, Kaczynski J. Waist-to-hip ratio but not body mass index predicts liver cirrhosis in women. Scandinavian Journal of Gastroenterology. 2018;53(2):212-7.
41. Schwartz LM, Persson EC, Weinstein SJ, Graubard BI, Freedman ND, Männistö S, et al. Alcohol consumption, one-carbon metabolites, liver cancer and liver disease mortality. PLoS One. 2013;8(10):e78156.
42. Serfaty L, Chazouilleres O, Poujol-Robert A, Morand-Joubert L, Dubois C, Chretien Y, et al. Risk factors for cirrhosis in patients with chronic hepatitis C virus infection: results of a case-control study. Hepatology. 1997;26(3):776-9.
43. Stroffolini T, Cotticelli G, Medda E, Niosi M, Del Vecchio-Blanco C, Addolorato G, et al. Interaction of alcohol intake and cofactors on the risk of cirrhosis. Liver International. 2010;30(6):867-70.
44. Trembling PM, Apostolidou S, Gentry-Maharaj A, Parkes J, Ryan A, Tanwar S, et al. Risk of chronic liver disease in post-menopausal women due to body mass index, alcohol and their interaction: a prospective nested cohort study within the United Kingdom Collaborative Trial of Ovarian Cancer Screening (UKCTOCS). BMC Public Health. 2017;17(1):603.
45. Tverdal A, Skurtveit S, Selmer R, Myhre R, Thelle D. Coffee and wine consumption is associated with reduced mortality from alcoholic liver disease: follow-up of 219,279 Norwegian men and women aged 30–67 years. Annals of Epidemiology. 2018;28(11):753-8.
46. Wiley TE, McCarthy M, Breidi L, McCarthy M, Layden TJ. Impact of alcohol on the histological and clinical progression of hepatitis C infection. Hepatology. 1998;28(3):805-9.
47. Yang L, Zhou M, Sherliker P, Cai Y, Peto R, Wang L, et al. Alcohol drinking and overall and cause-specific mortality in China: nationally representative prospective study of 220 000 men with 15 years of follow-up. International Journal of Epidemiology. 2012;41(4):1101-13.
48. Yi S-W, Hong J-S, Yi J-J, Ohrr H. Impact of alcohol consumption and body mass index on mortality from nonneoplastic liver diseases, upper aerodigestive tract cancers, and alcohol use disorders in Korean older middle-aged men: prospective cohort study. Medicine. 2016;95(39).
49. Yuan JM, Ross RK, Gao YT, Henderson BE, Yu MC. Follow up study of moderate alcohol intake and mortality among middle aged men in Shanghai, China. Bmj. 1997;314(7073):18-23.
